# The effect of water regulation on organic acids and rhizosphere microorganisms in the root system of drought-tolerant rice under Cd stress

**DOI:** 10.3389/fpls.2026.1943785

**Published:** 2026-09-17

**Authors:** Jiahui Cui, Chaoping Shan, Ruixiang Chen, Bo Li, Jianjun Chen

**Affiliations:** College of Resources and Environment, Yunnan Agricultural University, Kunming, Yunnan, China

**Keywords:** cadmium stress, microorganisms, organic acids, rice, water regulation

## Abstract

Rice (*Oryza sativa* L.) is the primary food crop in southern China, where the southwestern region is characterized by freshwater shortages and frequent seasonal droughts. Dryland rice cultivation serves as an important approach to addressing the conflict between rice production and water utilization. However, farmland cadmium (Cd) pollution in this area is significant, and dryland cultivation may alter the soil ecological environment, thereby affecting Cd uptake by rice and threatening safe rice production. Pot experiments on water regulation (long-term flooding and dryland treatment) of two dryland rice varieties “Yunjing 37” (YJ37) and “Luyin 46” (LY46) analyzed the effects of water regulation on the growth of roots and Cd accumulation under Cd stress; studying the correlation between root organic acids and rhizosphere microbial communities, the rhizosphere mechanism by which dryland rice varieties reduce Cd accumulation risk was revealed. The results showed that under dryland conditions, soil Eh increased significantly, and Cd content in roots increased significantly. Water regulation significantly affected the organic acids and microbial community structure of the two dryland rice varieties. Aerobic conditions significantly increased soil Eh and root Cd accumulation compared to continuous flooding. Furthermore, aerobic treatment reduced the root contents of tartaric, acetic, and malic acids by an average of 65.93%, 75.33%, and 31.85%, respectively, while decreasing rhizosphere microbial diversity (Chao1 and Shannon indices fell by 10.54% and 4.94%). Under dryland conditions, the dominant bacterial genera in the rice rhizosphere were *Sphingomonas* and *Gaiella*, which were significantly and positively correlated with the contents of malic acid, tartaric acid, and succinic acid in the roots. Under flooding conditions, the dominant bacterial genera in the roots of dryland rice were *Anaeromyxobacter*, *Pedosphaera*, etc. These dominant microorganisms help inhibit the activation of soil Cd and reduce the accumulation of Cd in rice. Water regulation affects the soil Cd form by changing low-molecular-weight organic acids, soil Eh, and microbial community structure, thereby affecting the absorption of Cd by dryland rice. Under cadmium contamination, water management for rice, combined with the cultivation of drought-resistant and low-Cd-accumulating varieties capable of directionally recruiting beneficial Cd-immobilizing bacteria, can provide a theoretical basis for the safe production of rice.

## 1 Introduction

Rice (*Oryza sativa* L.), as one of the main food crops globally, consumes a large amount of agricultural freshwater resources in the traditional flooding irrigation process. According to predictions by the International Food Policy Research Institute, by 2050, the available freshwater resources are expected to decrease by 50% (Gupta et al., 2020), and climate change will lead to a 20% decrease in rice production in developing countries, thereby threatening global food security (Jiang et al., 2025a). Transplanting rice remains the main planting method globally, consuming 45% of the world’s freshwater resources, about two to three times that of other crops (Narwal et al., 2025). Seasonal drought has led to increasingly tight water resources, becoming the main limiting factor for global rice production (Debabrata et al., 2021), and dryland rice technology is gradually developing. Aerobic rice technology employs direct seeding under non-flooded conditions, relying primarily on natural rainfall supplemented by targeted irrigation during critical growth stages (Jiang et al., 2025b). Requiring only one-third of the water used in traditional flooded systems (Khepar et al., 2000), aerobic rice offers a sustainable solution for water-scarce regions (Ma et al., 2025).

In the process of rice dryland cultivation, soil heavy metal toxicity, particularly cadmium (Cd) pollution, poses a severe challenge to food safety. The “National Soil Pollution Status Survey Report” jointly released by the Ministry of Environmental Protection and the Ministry of Land and Resources in 2014 shows that the Cd pollution exceedance rate is 7.0% (Zhao et al., 2021). The sources of Cd pollution in paddy soil mainly include human activities, mining and metallurgy, industrial production, wastewater irrigation, and secondary enrichment in soil formation processes (Zhang et al., 2014). The southwest region of our country has a higher cadmium background value (Xia et al., 2022). The accumulation of Cd in rice can lead to reduced plant height, reduced tillering number, reduced biomass of seeds and roots, reduced concentration of essential elements, and inhibition of growth (Gao et al., 2018). Changes in soil redox potential (Eh), organic acids, and micro-ecological environment affect the availability of Cd, thereby posing risks to the safe production of rice.

Plant root systems release a large amount of metabolites into the rhizosphere, including low-molecular-weight organic acids, which can change the pH of rhizosphere soil and form metal-organic acid complexes with metal ions (Chen et al., 2017). Low-molecular-weight organic acids directly affect the root environment to drive changes in the rhizosphere microbial community (Liu et al., 2007; Badri et al., 2013), helping plants to recruit specific beneficial microbial communities to directly or indirectly help plants resist Cd stress. Plants can dynamically regulate the formation of microbial communities associated with themselves by changing the composition of root exudates, enhancing the recruitment of beneficial microorganisms (Zhu et al., 2024), and these beneficial microorganisms induce resistance mechanisms within the plant to protect it from Cd damage.

Water regulation significantly affects the community structure of rhizosphere microorganisms in rice. Water regulation further regulates microbial function by altering the soil redox environment (Zang et al., 2023). Drought stress alters the composition of the rhizosphere microbiome, leading to the enrichment of drought-adapted bacterial groups, particularly an increase in the relative abundance of *Actinobacteria* and a decrease in *Proteobacteria* (Xu et al., 2018b). Water regulation influences the composition of microbial communities. Without flooding, microbial hubs are completely transformed into aerobic microorganisms, while *Actinobacteria* and *Chloroflexi* become the most sensitive to flooding, with *Chloroflexi* numbers increasing under dryland conditions (Cui et al., 2019). The dynamics of the microbiome under different water status characteristics (persistent flooding and dryland conditions) are crucial, as changes in water conditions promote the dominance of aerobic and anaerobic microorganisms in different environments. Cd stress significantly affects the composition of bacterial communities in rice rhizosphere soil. Under Cd stress, significantly altered microbial communities are typically categorized as Cd-resistant and Cd-sensitive types (Liu et al., 2022). *Proteobacteria* are often reported to be dominant in Cd-rich environments, recruited by rice root exudates as potential participants in plant tolerance and detoxification processes (Li et al., 2024; Lin et al., 2025). *Acidobacteria* are the dominant bacterial groups in soils of high-concentration heavy metal mining areas, indicating their potential heavy metal resistance and adaptability (Liu et al., 2022). Current research predominantly focuses on either the effects of water management on rice growth or the responses of the rice rhizosphere microbiome under Cd stress. However, studies on the impacts on rice growth, rhizosphere soil environment, and microbial communities driven by the dual factors of Cd stress and water management remain insufficient. Therefore, against the background of Cd stress, this study established a water management experiment to analyze the response characteristics of rice growth, rhizosphere organic acids, and microorganisms, aiming to elucidate the synergistic response mechanism of the rhizosphere soil microbial community in dryland rice to the combined effects of water management and Cd stress.

## 2 Materials and methods

### 2.1 Test materials

The soil sample was collected from the 0~20 cm cultivated soil in Jinning District, Yunnan Province (longitude 102°27′53″, latitude 24°35′15″), sieved (2 mm) for reserve. Its physicochemical properties are as follows: pH: 4.99, organic matter: 13.33 g·kg-1, total nitrogen: 0.39 g·kg^-1^, total phosphorus: 1.37 g·kg^-1^, total potassium: 1.16 g·kg^-1^, alkali-hydrolyzed nitrogen: 0.043 g·kg^-1^, available phosphorus: 3.64 mg·kg^-1^, available potassium: 99.50 mg·kg^-1^, soil Cd content: 0.81 mg·kg^-1^. The planting materials were selected from 2 drought-tolerant rice varieties screened in the early stage by the research group: “Yunjing” 37 (YJ37) and “Luyin 46” (LY46). “Yunjing 37” is a rice variety suitable for dryland cultivation, and “Luyin 46” is a drought-resistant rice variety, with seeds sourced from the Rice Research Institute of Yunnan Academy of Agricultural Sciences.

### 2.2 Test design

To study the effect of water management on the rhizosphere microbial community of dryland rice, pot experiments were conducted with two dryland rice varieties planted under different water control conditions, including long-term flooding (field water content 100%, 100% WHC) and dryland treatment (field water content 60-70%, 60-70% WHC). The soil Cd pollution level in the experiment was set at 10 mg·kg-1, with 3 repetitions, totaling 12 pots. This study mainly focuses on the effect of water control on the rhizosphere microbial community of dryland rice under Cd stress, referring to the rich mineral resources in some parts of Yunnan Province, where the waste slag and wastewater from ore dressing cause high Cd content in cultivated soil, such as the surrounding farmland soil Cd content in Huize County, Yunnan Province, which is 9.07 mg·kg^-1^ (Wu et al., 2018), and select a soil Cd pollution level of 10 mg·kg^-1^.

Add CdCl_2_·2.5H_2_O solution to the soil to prepare Cd-polluted soil, and reserve it after balancing for 15 days. Select white, vigorous rice seeds for sowing into plastic pots (inner diameter 27.5 cm, height 24 cm) containing the test soil for the experiment. Each plastic pot contains 5 kg of soil, with 10 rice seeds sown per pot, and 4 plants retained at the three-leaf stage. The day before transplanting, mix urea, potassium dihydrogen phosphate, and potassium oxide in a 1:1:1 (mass ratio) and apply it as bottom fertilizer. Apply 1 g of urea as topdressing during the tillering stage. Under dryland conditions, maintain soil moisture content at 60-70% of the field’s maximum water-holding capacity using a weighing-watering method and a water potential gauge. Water when the pressure of the water potential gauge drops to -30 kPa, and calculate the amount of water using the weighing-watering method. Under continuous flooding conditions, maintain a 2 cm water layer on the soil surface. The temperature during the experiment was 28 ± 2°C.

### 2.3 Sample collection

Harvest rice plants at the maturity stage. Collect rhizosphere soil of rice plants, store it at -80°C in a refrigerator, and use it for metagenomic sequencing of microorganisms. After harvesting rice plants, wash the plants with deionized water; part of which is used to collect low-molecular-weight organic acids from the roots, and part of the roots is scalded at 105°C for 0.5 h and then continuously dried at 75°C until constant weight, ground, and reserved.

### 2.4 Index determination

#### 2.4.1 Soil redox potential measurement

During the rice harvest period, the redox potential meter (MORP30, Keli) is used to *in situ* measure the Eh at a soil depth of 5 cm in the rhizosphere region of rice.

#### 2.4.2 Determination of Cd content in rice roots

Weigh 0.3000 g of the sample dried and ground to a powder, place it in a conical flask, add 10 mL of a HNO_3_-HClO_4_ mixed solution (9 + 1), cover and soak overnight, cover with a bent neck funnel and digest on a graphite furnace electric hot plate, if it turns brown-black, add HNO_3_ until white smoke appears, the digestion solution becomes colorless and transparent or slightly yellow, cool it down, then transfer the digestion solution into a 50 mL volumetric flask, wash the conical flask with deionized water 3 times, merge the washings into the volumetric flask, dilute to volume with deionized water, mix well, and wait for measurement.

#### 2.4.3 Determination of low-molecular-weight organic acids in rice

Harvest complete rice plants at maturity, randomly select 1 hole from each pot, immediately return to the laboratory, wear gloves to gently remove the rhizospheric soil adhering to the roots, then rinse the rice roots with ionized water, place the entire rice root system into a collection bottle containing 200 mL of foil-wrapped solution, extract and collect low-molecular-weight organic acids for 24 h, filter the collected liquid through a 0.45 µm filter membrane, concentrate to 10 mL at 40 °C in a vacuum rotary evaporator, freeze at -20 °C for storage and use. Before analysis, filter the samples with a 0.45 µm microporous filter membrane to remove impurities. The determination of organic acid content is carried out by HPLC, obtaining the chromatogram of a mixture of five organic acids (succinic acid, tartaric acid, oxalic acid, acetic acid, and malic acid) standard samples under selected chromatographic conditions, thereby determining the content of low-molecular-weight organic acids in the sample.

#### 2.4.4 Rice rhizosphere soil microbial metagenome sequencing

Based on the completion of Shanghai Meiji Biopharmaceutical Technology Co., Ltd., Soil DNA extraction and quality control: Take root rhizosphere soil samples stored at -80°C and extract DNA using the FastPure Soil DNA Isolation Kit (Magnetic bead) (MJYH, Shanghai, China). After detection as qualified using NanoDrop 2000, Qubit 4.0, and 1% agarose gel electrophoresis, use Covaris M220 to fragment to about 350 bp. Construct PE libraries using the NEXTFLEX^®^ Rapid DNA-Seq Kit (Bio-Rad Scientific, USA). After library qualification, perform metagenomic sequencing based on the Illumina NovaSeqTM X Plus (Illumina, USA) platform. Use fastp (version 0.20.0) to trim adapter sequences at the 3’ and 5’ ends of reads; use fastp (version 0.20.0) to remove reads shorter than 50 bp and with an average base quality value below 20 after trimming, retaining high-quality sequences.

Concatenation assembly and gene prediction: Use MEGAHIT (version 1.1.2) to optimize assembly sequences and screen for contigs s ≥300 bp. Use Prodigal (version 2.6.3) to predict ORFs. Select genes with nucleotide lengths ≥100 bp for translation into amino acid sequences.

Non-redundant gene construction: Use CD-HIT (version 4.7) to cluster gene sequences (90% identity, 90% coverage), and construct a non-redundant gene set by taking the longest sequence. Use SOAPaligner software (version soap2.21 release) to map high-quality reads back to the gene set (95% identity) and calculate gene abundance.

Species and function annotation: Use Diamond (version 2.0.13) to map non-redundant genes to the NR database (e-value ≤ 1e-5). Obtain species annotation information. Calculate the abundance of corresponding functional categories using the total abundance of genes corresponding to KO and Pathway.

### 2.5 Statistical analysis

Data were analyzed using Microsoft Excel 2019, and the significance of differences was tested using IBM SPSS Statistics 27.0 software (independent-samples t-test, P<0.05), and data plotting was performed using Origin 2021. The species annotation of microorganisms was based on the cloud service provided by Shanghai Majorbio Biopharm Technology Co., Ltd. (https://www.i-sanger.com), and the amino acid sequences of non-redundant gene sets were compared to NR (species annotation). Alpha diversity indices were calculated for species abundance at the phylum level of metagenomes, and inter-group comparisons were made. Beta diversity analysis was conducted. Comparisons between data groups were made using one-way ANOVA (One-way ANOVA) and the Kruskal-Wallis H test.

## 3 Results and analysis

### 3.1 The effect of water regulation on the growth of low-Cd accumulating dryland rice

Rice sensitivity to cadmium is related to cultivation varieties. ‘Yunjing 37’ has a good, high, and stable yield, strong resistance to rice blast, and cold tolerance. ‘Luyin 46,’ as a drought-resistant variety, has a compact plant type and strong tillering ability, taking into account the characteristics of both multi-spike and large-spike types. Both rice varieties show good growth adaptability under conventional flooding conditions and drought conditions. Under the influence of a cadmium content of 10 mg·kg^-1^, different water conditions and varieties have a certain impact on rice biomass. Under flooding conditions, the root length, root tip number, and root surface area of ‘LY46’ are all lower than those of ‘YJ37’, with significant differences in root length. Under dryland conditions, the root volume of ‘LY46’ is significantly different from that of ‘YJ37’, increasing by 57.35% (**Figure 1**). Under Cd stress, aerobic treatment exerted a more pronounced inhibitory effect on rice root volume, root tip count, and root surface area than flooded treatment.

**Figure 1:**
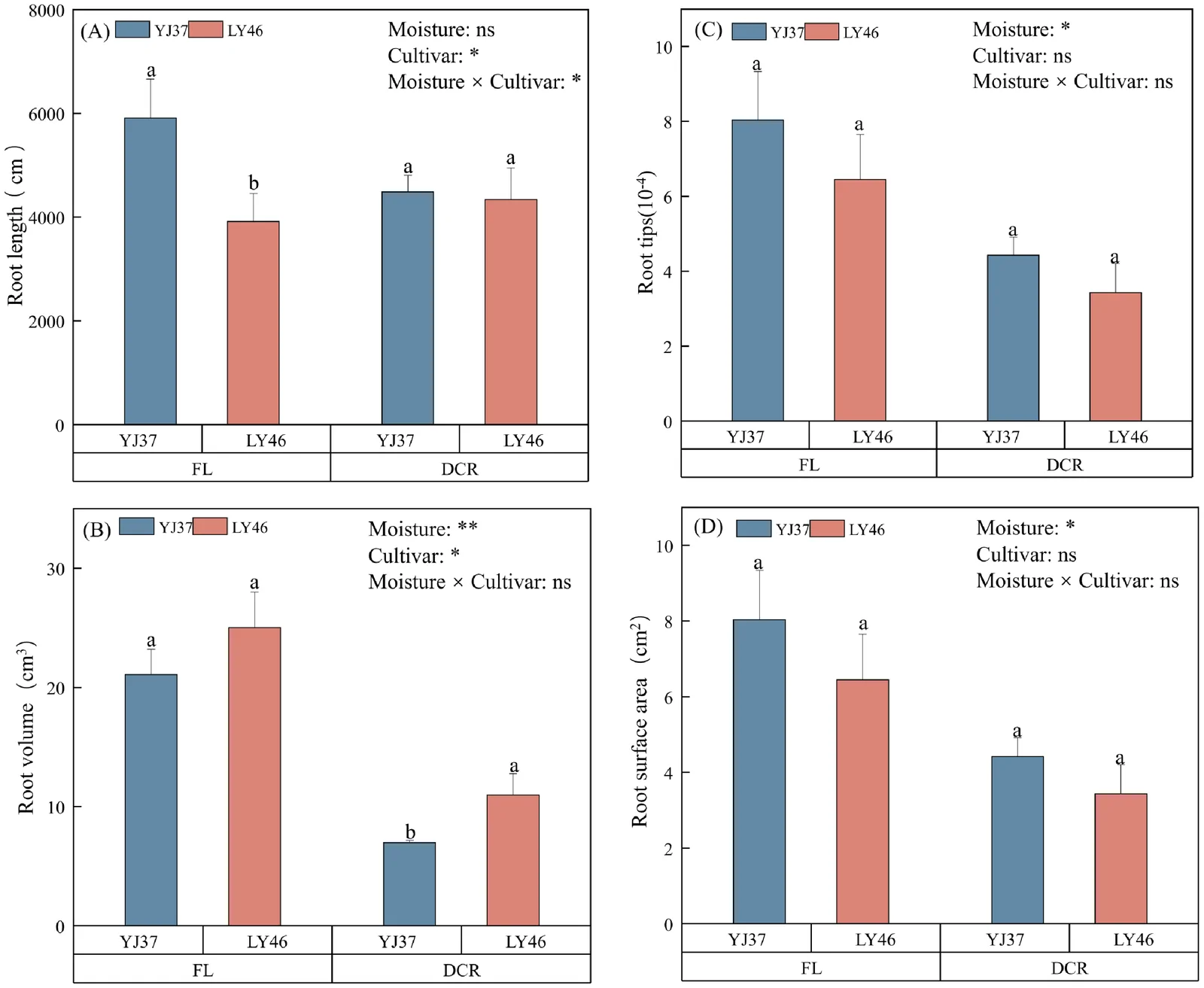
Effects of water regulation on growth parameters of low Cd accumulation dryland rice cultivation: **(A)** Root length, **(B)** Root volume, **(C)** Root tip number, **(D)** Root surface area. Different letters indicate significant differences in dryland rice biomass (*P* < 0.05); FL represents the flooding condition (100% WHC), DCR represents the dryland condition (60-70% WHC); ‘YJ37’ represents ‘Yunjing 37’, ‘LY46’ represents ‘Luyin 46’.

### 3.2 Response of soil Eh and secretion of low-molecular-weight organic acids by roots under cadmium stress to water regulation

#### 3.2.1 The effect of water regulation on the redox potential of soil in dryland rice cultivation and the cadmium content in roots

Under Cd stress (10 mg·kg^-1^), water regulation significantly affects the Eh of the two dryland rice soils (**Figure 2A**). Compared with flooding conditions, soil Eh increases under dryland conditions. Under flooding conditions, there are significant differences in Eh between the two dryland rice soils (*P* < 0.05), with the Eh of ‘YJ37’ being 0.84 times that of ‘LY46’, and Eh showing a positive correlation with root Cd concentration (R^2^ = 0.908, *P* < 0.01). Water regulation significantly affects the Cd content in the roots of dryland rice (*P* < 0.05) (**Figure 2B**). Compared with flooding conditions, the dryland condition significantly increases the Cd content in the roots of rice, with a significant difference in root Cd content between ‘YJ37’ and ‘LY46’, differing by a factor of 1.38. Under flooding conditions, sulfate is reduced to S^2−^ and reacts with Cd^2+^ to form insoluble sulfides, which is consistent with the lower root cadmium content under flooding conditions (**Figure 2B**). An increase in Eh promotes the desorption of cadmium from iron oxide minerals, while cadmium sulfide is oxidized and decomposed to release free Cd²^+^, leading to an increase in cadmium content in the roots of rice under dryland treatment.

**Figure 2:**
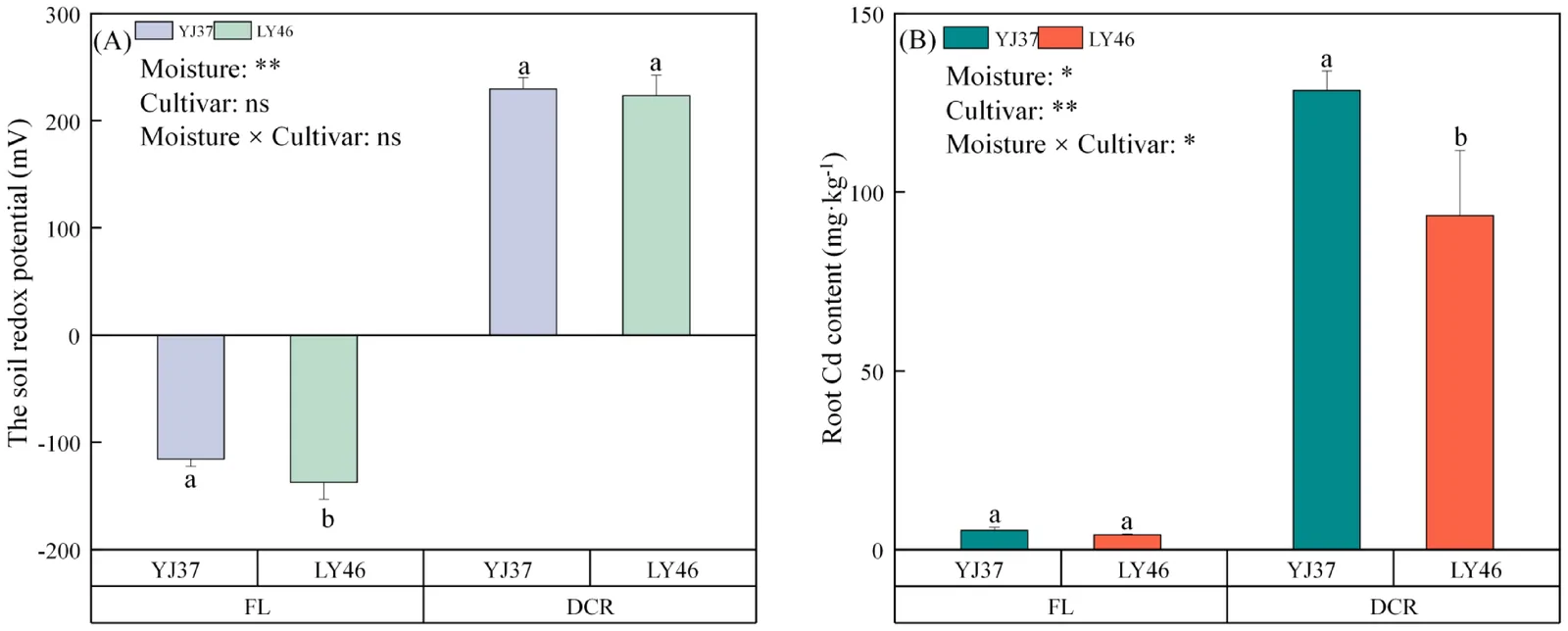
**(A)** Effects of water regulation on Eh of soil in low Cd accumulation dryland rice cultivation, **(B)** Effects on Cd content in rice roots. Different letters indicate significant differences in dryland rice biomass (*P* < 0.05); FL represents the flooding condition (100% WHC), DCR represents the dryland condition (60-70% WHC); ‘YJ37’ represents ‘Yunjing 37’, ‘LY46’ represents ‘Luyin 46’.

### 3.3 The effect of water regulation on the content of low molecular weight organic acids in rice

One of the important mechanisms by which plants alleviate soil heavy metal toxicity is the secretion of organic acids. Under Cd stress (10 mg·kg^-1^), water regulation significantly affects the content of low molecular weight organic acids in the roots of dryland rice (*P* < 0.05) (**Figure 3**). Under flooding conditions, there were no differences in the contents of succinic acid, tartaric acid, and oxalic acid in the roots of dryland rice. Under flooded conditions, a significant difference was observed in the malic acid content between the two dryland rice cultivars (*P* < 0.05), with ‘LY46’ exhibiting a significantly higher malic acid content that was 1.36 times that of ‘YJ37’. Under aerobic conditions, the root contents of succinic acid, tartaric acid, malic acid, and oxalic acid in ‘LY46’ were significantly elevated, being 2.89, 1.81, 1.55, and 0.85 times higher than those in ‘YJ37’, respectively. Under the same water regime, no significant difference was found in root acetic acid content between the two cultivars. Specifically, under aerobic conditions, ‘LY46’ displayed significantly higher contents of succinic, tartaric, malic, and oxalic acids compared to ‘YJ37’.The organic acids secreted in the rhizosphere can efficiently chelate heavy metals, alleviate their toxicity to the roots, and cause the cadmium content in the roots of ‘LY46’ to be lower than that in ‘YJ37’ under drought conditions. This indicates that ‘LY46’ can actively excrete specific organic acids, chelate free Cd²^+^ extracellularly, and thus constitutes a species-specific chemical detoxification mechanism.

**Figure 3:**
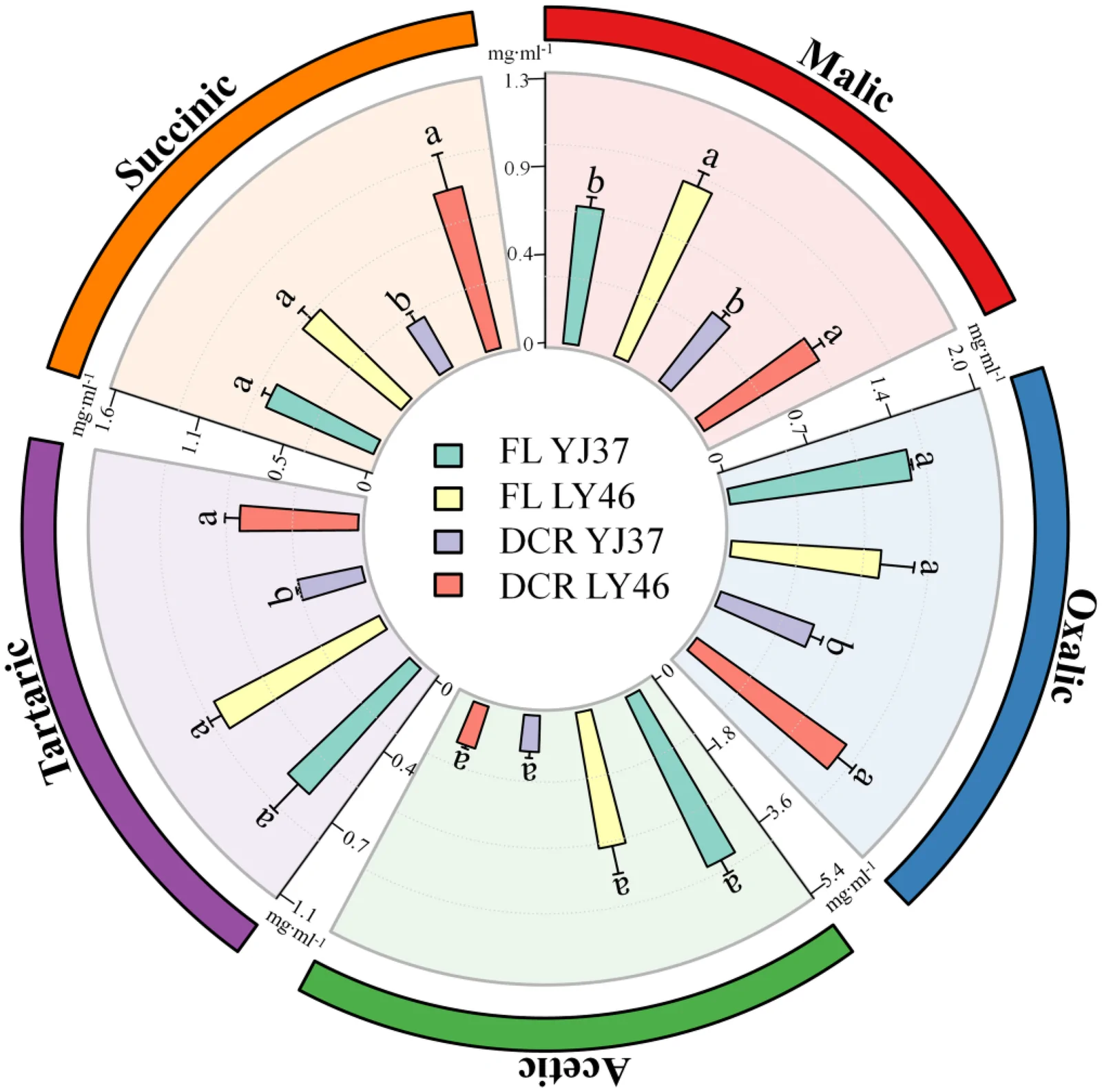
Effects of water regulation on low-molecular-weight organic acids in the root system of dryland rice. Different letters indicate significant differences in dryland rice biomass (*P* < 0.05); FL represents the flooding condition (100% WHC), DCR represents the dryland condition (60-70% WHC); ‘YJ37’ represents ‘Yunjing 37’, ‘LY46’ represents ‘Luyin 46’.

### 3.4 The effect of water regulation on the rhizosphere microbial community of rice

#### 3.4.1 The effect of water regulation on the composition of the soil microbial community structure of rice

To evaluate the similarities and differences in the composition of soil microbial communities under different treatments, under Cd stress (10 mg·kg^-1^), the results of principal coordinate analysis (PCoA) of the rhizosphere soil microbial community in rainfed rice under moisture regulation indicated significant differences in community structures among different groups (**Figure 4A**). Under different water regulation conditions, the samples are obviously separated on the PC1 axis, indicating that there are significant differences in the microbial community structure between dryland and flooded treatments. The distance between ‘YJ37’ and ‘LY46’ is relatively far, and the species composition differs significantly. Under different water regulation conditions, ‘YJ37’ and ‘LY46’ are separated from each other. This indicates that the ANOSIM test shows significant differences between groups (*P* < 0.005). Water regulation is an important factor driving the differentiation of the rhizosphere microbial community structure under Cd stress, and different water conditions significantly affect the composition of the rhizosphere microbial community, indicating that rhizosphere microbes are relatively sensitive to water conditions. This phenomenon may be related to the physiological activities of the roots under different water conditions, such as the release of root exudates.

**Figure 4:**
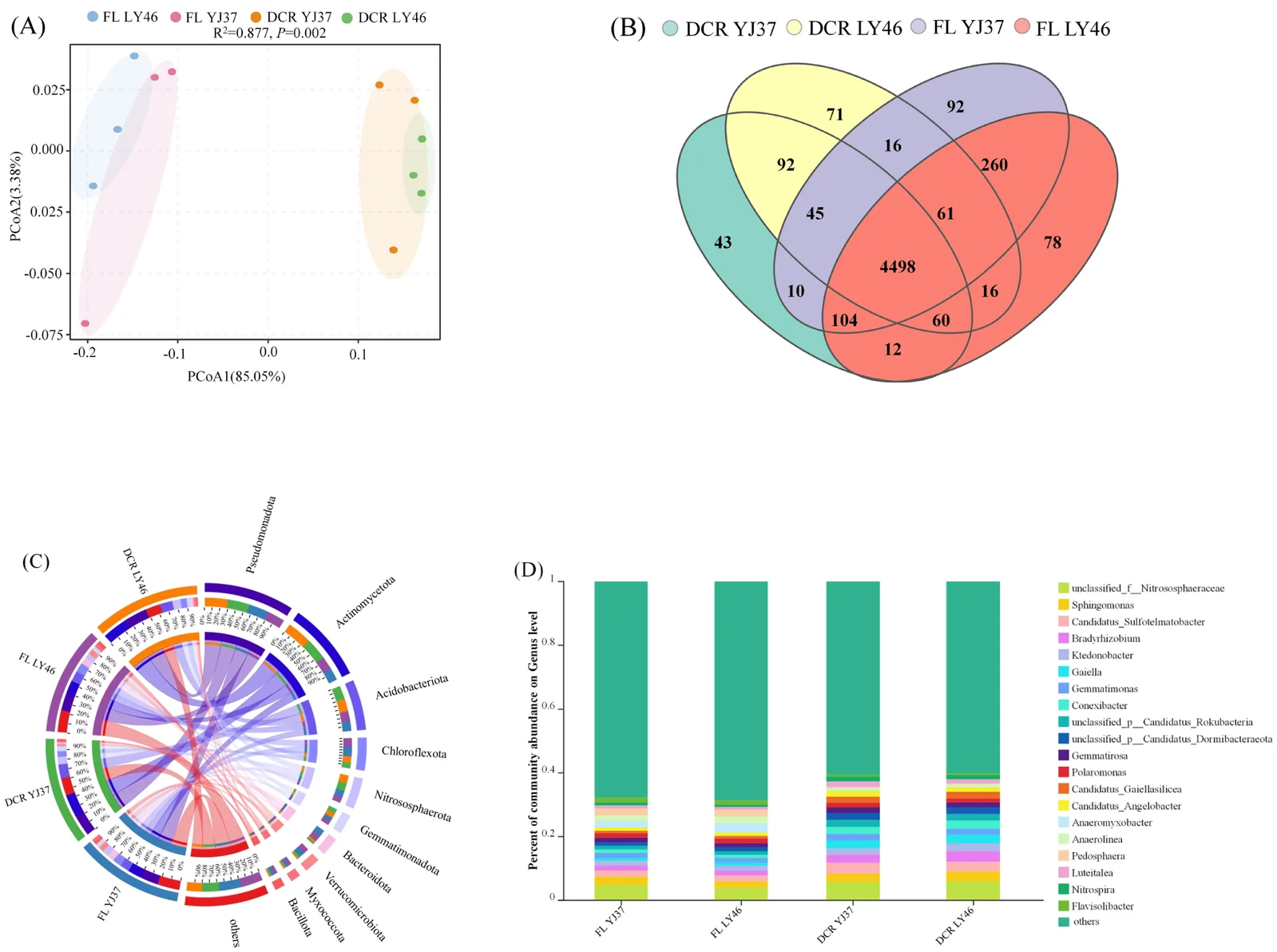
Beta diversity: **(A)** Principal Coordinate Analysis (PCoA) **(B)** Venn diagram. Root rhizosphere bacterial community species composition and relative abundance analysis: **(C)** Circos sample and species relationship; **(D)** Root rhizosphere bacterial genus-level classification histogram.

Using Venn diagrams to analyze the composition of unique clusters, the degree of overlap of common clusters, and the similarity of soil microbial communities under different treatment conditions. At the genus level, under water control conditions, the common clusters of rhizosphere microorganisms in dry rice reached 4498 genera, accounting for 82.41% of the total clusters; among them, the unique clusters of the dry treatment were 92 genera, accounting for 1.69%; and the unique clusters of the flooding treatment were 260 genera, accounting for 4.76%(**Figure 4B**). This indicates that the flooding environment has constructed a more complex and more effective microbial barrier for Cd control. Under dry conditions, the community structure is single in the oxidizing environment with high Cd, leading to an increase in cadmium content in the roots under dry conditions. Although the rhizosphere microbial communities under dry and flooding treatments share a highly conserved core cluster, there is still a certain proportion of specific clusters, indicating that different water conditions can significantly affect the species composition and structural differentiation of rhizosphere microbial communities.

Through the Circos map (**Figure 4C**), dominant bacterial genera at the gate level were identified. Under flooding conditions, *Actinomycota*, *Pseudomonadota*, and *Acidobacteriota* were the main bacterial communities, accounting for 22% - 25%, 20% - 23%, and 14%, respectively. Under dryland conditions, *Pseudomonadota* dominated, while *Actinomycota* and *Acidobacteriota* showed a significant decrease in proportion compared to flooding conditions, resulting in a significant structural shift in the community.

Under dryland conditions, aerobic and facultative anaerobic bacteria are significantly enriched in rhizosphere microorganisms, with dominant genera including unclassified_f_*Nitrososphaeraceae*, Candidatus_*Solibacter*, *Sphingomonas*, *Bradyrhizobium*, *Gaiella*, and *Conexibacter* (**Figure 4D**). Under dryland treatments, plants may selectively recruit the aforementioned cadmium-tolerant aerobic bacteria by secreting large amounts of carbon and nitrogen sources. In contrast, under flooding conditions, although the aforementioned dominant genera still occupy a certain proportion, the abundance of anaerobic microorganisms such as *Anaeromyxobacter*, *Pedosphaera*, and *Anaerolinea* is higher than that in the dryland group. These bacterial populations can adapt to the reductive environment caused by flooding and use nitrates or iron-manganese oxides as alternative electron acceptors to complete metabolism. Under dryland conditions, changes in Eh shift the microbial community toward aerobic and facultative anaerobic bacteria. Under dryland conditions, cadmium is released through the release of iron-manganese (hydroxide) oxides or the oxidation of sulfides, resulting in significant enrichment of cadmium-tolerant microbial communities such as *Sphingomonas* and *Bradyrhizobium*. The structural changes are directly related to the increased absorption of Cd by rice roots.

#### 3.4.2 The effect of water regulation on the differential diversity of soil microbial communities in rice plants

The Chao1 index is a core α diversity index used in ecology and microbiome studies to estimate the total number of species in the community. The Shannon index is applied to measure species diversity, providing insights into the evenness and distribution of different species in the sample, while the Simpson index reflects the concentration of the community. Under Cd stress (10 mg·kg^-1^), water regulation significantly affects the diversity of soil microorganisms in dryland rice (*P* < 0.05) (**Figure 5**). Under dryland conditions, the Chao1 index and Shannon index of soil microorganisms in the rhizosphere of dryland rice decreased by 10.54% and 4.94%, respectively, while the Simpson index increased by 64.61%. This indicates that dryland rice cultivation may significantly affect the richness and diversity of the rhizosphere soil microbial community, screening out a few cadmium-tolerant dominant bacterial populations, such as potential Cd immobilization bacteria, with a decrease in community species evenness and a decline in overall richness and diversity.

**Figure 5:**
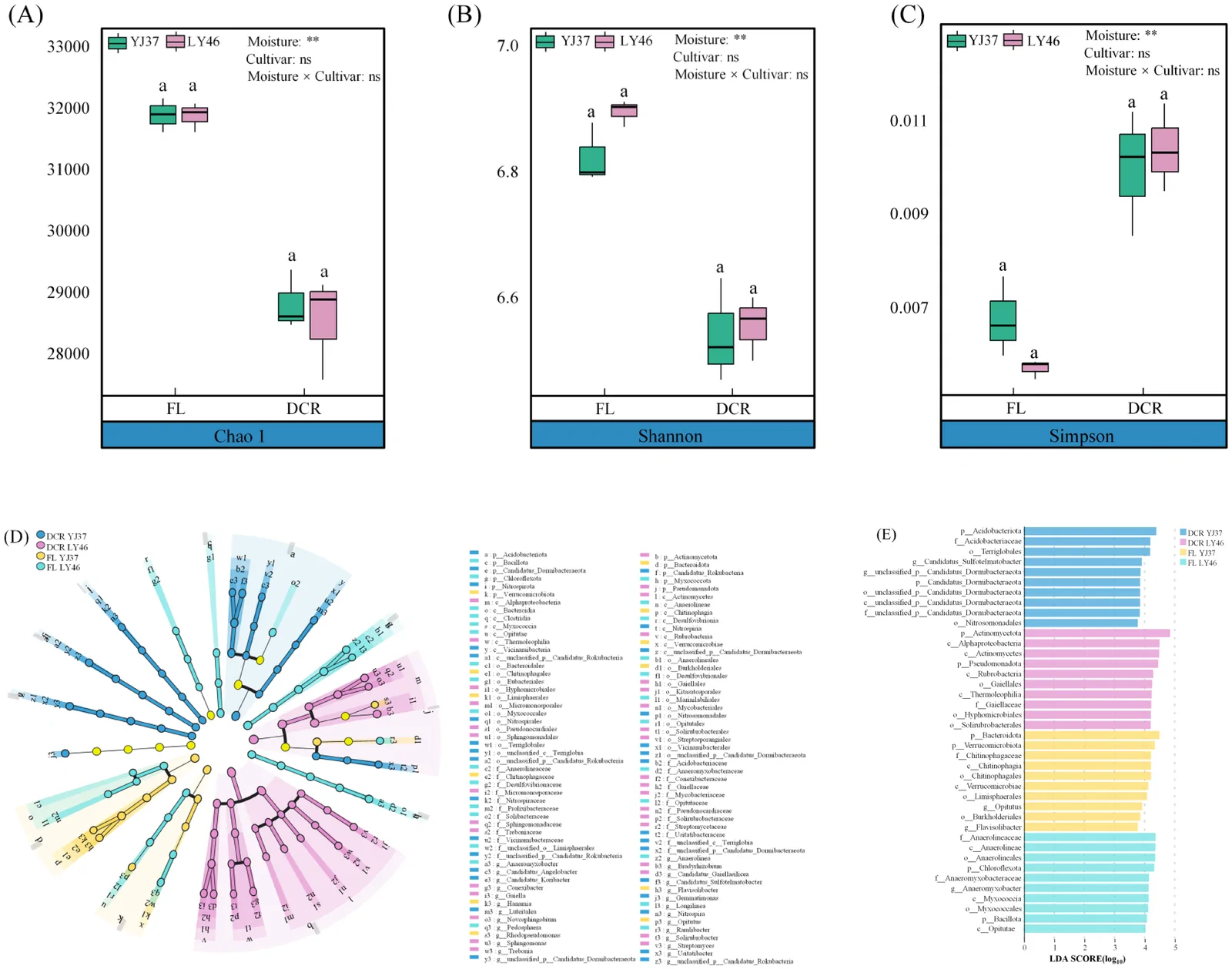
Alpha diversity index: **(A)** Chao 1 index, **(B)** Shannon index, **(C)** Simpson index. LEfSe analysis: **(D)** LEfSe evolutionary branch diagram, **(E)** LDA score histogram.

Under flooding conditions, ‘YJ37’ enriched the *Bacteroidota*, *Verrucomicrobiota*, and *Actinomycetota*, while ‘LY46’ enriched the *Chloroflexota*, *Bacillota*, and *Nitrospirota*. Under dryland conditions, ‘YJ37’ enriched the *Acidobacteriota*, Candidatus_*Dormibacterota*, and *Chloroflexota*, while ‘LY46’ enriched the *Actinomycota*, *Pseudomonadota*, and *Cyanobacteriota* (**Figure 5D**). Based on the LDA scores (**Figure 5E**), bacterial biomarkers for each of the four treatments were identified. Under flooding conditions, the biomarkers for ‘YJ37’ were p_*Bacteroidota*, p_*Verrucomicrobiota*, f_*Chitinophagaceae*, and for ‘LY46’ were f_*Anaerolineaceae*, c_*Anaerolineae*, o_*Anaerolineales*. Under dryland conditions, the biomarkers for ‘YJ37’ were p_*Acidobacteriota*, f_*Acidobacteriaceae*, o_*Terriglobales*, and for ‘LY46’ were p_*Actinomycetota*, c_*Alphaproteobacteria*, c_*Actinomycetes*. The biomarkers under dryland conditions reflect typical aerobic rhizosphere characteristics, while the biomarkers under flooding conditions (especially the enrichment of *Chloroflexota*) reflect the reductive characteristics of flooded soils. The enrichment of biomarkers under flooding conditions indicates that under anaerobic conditions, microorganisms may maintain low Cd availability through their anaerobic metabolic processes, reducing the uptake of Cd by roots. The enrichment of biomarkers under dryland conditions is a result of rice actively recruiting them to respond to the high toxicity of Cd in the oxidative environment, to assist the roots in resisting Cd stress.

#### 3.4.3 The effect of water regulation on the functional annotation and metabolic pathways of soil microorganisms in rice

Based on the Cluster of Orthologous Groups of proteins) COG, the gene sequences of each treatment group were annotated for function (**Figure 6A**) and were classified into 25 functional categories. The metabolic category (C, G, E, F, H, I, P, Q) dominated the four core categories (metabolic, cellular processes and signaling, information storage and processing, and functionally unclassified), accounting for 44.31% to 45.88%. Within the metabolic category, the genes with the highest abundance were E (amino acid transport and metabolism), G (carbohydrate transport and metabolism), and C (energy production and conversion), indicating that this soil microbial community possesses extremely strong functional potential in energy transformation, the carbon-nitrogen cycle, and basic substance metabolism. Among them, the metabolic category had a higher proportion than the waterlogging treatment in the dryland treatment, and the cellular processes and signaling category occupied a secondary position, with a proportion range of 26.99% to 28.53%. The proportion of dryland conditions was reduced by 1.11% to 1.54% compared to waterlogging conditions, among which T (signal transduction mechanism) and M (cell wall/membrane/envelope biogenesis) were the two core groups with the highest proportion. Under waterlogging conditions, the abundance was all above 7.30%, while under dryland conditions, it decreased by 0.45% to 0.47%. This indicates that changes in water conditions cause the transformation of microbial functions. Under waterlogging conditions, the high abundance of signal transduction (T) and membrane structure biogenesis (M) functions reflects that microorganisms can more actively maintain complex interactions among microbial communities in a reducing environment and assist in the precipitation or control of Cd by constructing stable membrane structures. Under dryland conditions, a higher proportion of the metabolic category indicates that the microbial community resists cadmium stress by strengthening basic substance metabolism. It also indicates the richness and diversity of the microbial community under waterlogging conditions.

**Figure 6:**
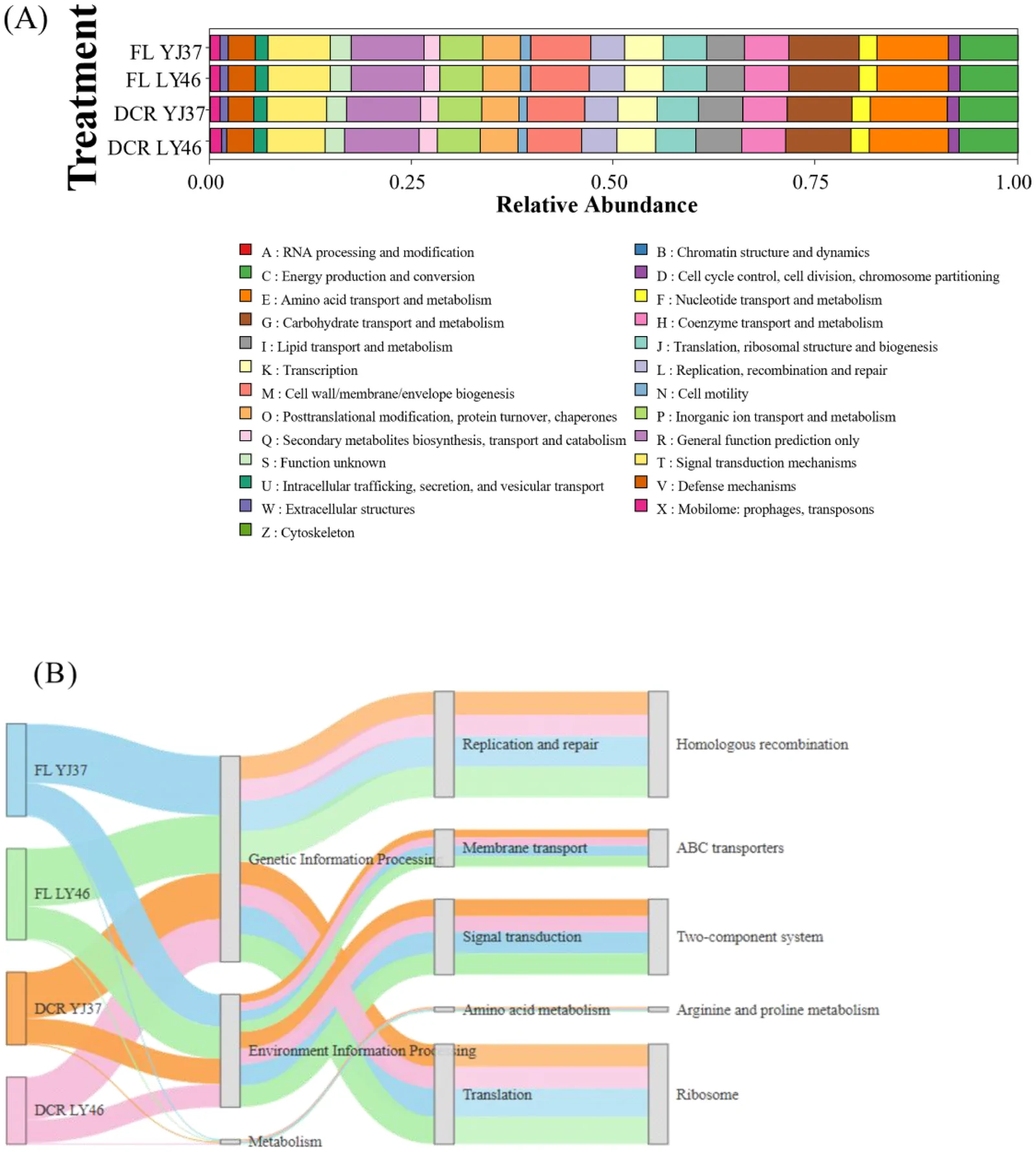
Functional and metabolic pathway analysis: **(A)** COG functional annotation, **(B)** KEGG third-level metabolic pathway analysis.

The KEGG pathway prediction Sankey diagram shows the data flow of the most abundant key pathways in each sample. The functional enrichment analysis selects and displays the most core functional modules, including 3 primary categories (Genetic Information Processing, Environment Information Processing, Metabolism), 5 secondary categories (Replication and repair, Membrane transport, Signal transduction, Translation, Amino acid metabolism), and 5 tertiary pathways (Homologous recombination, ABC transporters, Two-component system, Ribosome, Arginine and proline metabolism) for the flow of each treatment group (**Figure 6B**). At the primary category level, Genetic Information Processing and Environmental Information Processing, as the widest receiving nodes, converge all the input of the treatment groups, and the connecting lines are generally wide, indicating that the function of maintaining genetic information and environmental adaptation of the samples remains highly active under different treatments. Ribosome and Homologous recombination mainly receive inputs from the waterlogging treatment, and the connecting line width is obvious, indicating that the microbial community is in an active proliferation and vigorous metabolic period. Under dryland conditions, the two-component system and ABC transporters function is significantly enhanced. This indicates that under the Cd activation pressure caused by dryland farming, microorganisms actively adjust their physiological behavior by enhancing detoxification and environmental perception functions to resist heavy metal toxicity.

### 3.5 The interaction between root organic acids and microorganisms in dryland rice under water regulation

A connection between bacterial communities and root-associated low-molecular-weight organic acids was established through Procrustes analysis and RDA (redundancy analysis). Procrustes analysis results showed a significant overall correlation between bacterial communities and low-molecular-weight organic acids, with a low M^2^ value and a significant P-value (M^2^ = 0.338, *P* = 0.003) (**Figure 7A**). To further illustrate the relationship between organic acids and major microorganisms, RDA analysis elucidated the relationship between the top five most abundant microorganisms in the rhizosphere of dryland rice and low-molecular-weight organic acids. The RDA1 axis and RDA2 axis collectively explained 80.00% of the variables (**Figure 7B**). Malic acid, oxalic acid, tartaric acid, and succinic acid exhibited a significant positive correlation with *Bradyrhizobium* and *Sphingomonas*, and a negative correlation with g_Candidatus_*Sulfitelmatobacter*. Acetic acid content was positively correlated with g_unclassified_f_*Nitrososphaeraceae* and negatively correlated with *Gaiella* and g_Candidatus_*Sulfitelmatobacter*. Under cadmium stress, rice can release specific low-molecular-weight organic acids (such as oxalic acid and succinic acid) as signaling molecules or excretory carbon sources to selectively recruit and enrich beneficial bacteria such as *Sphingomonas* and *Bradyrhizobium* to resist cadmium stress. The enriched beneficial bacteria can help retain cadmium in the rhizosphere or on the root surface iron film by secreting extracellular polymers or chelating transformations, thereby effectively inhibiting the transport of toxic heavy metals to the upper part of rice.

**Figure 7:**
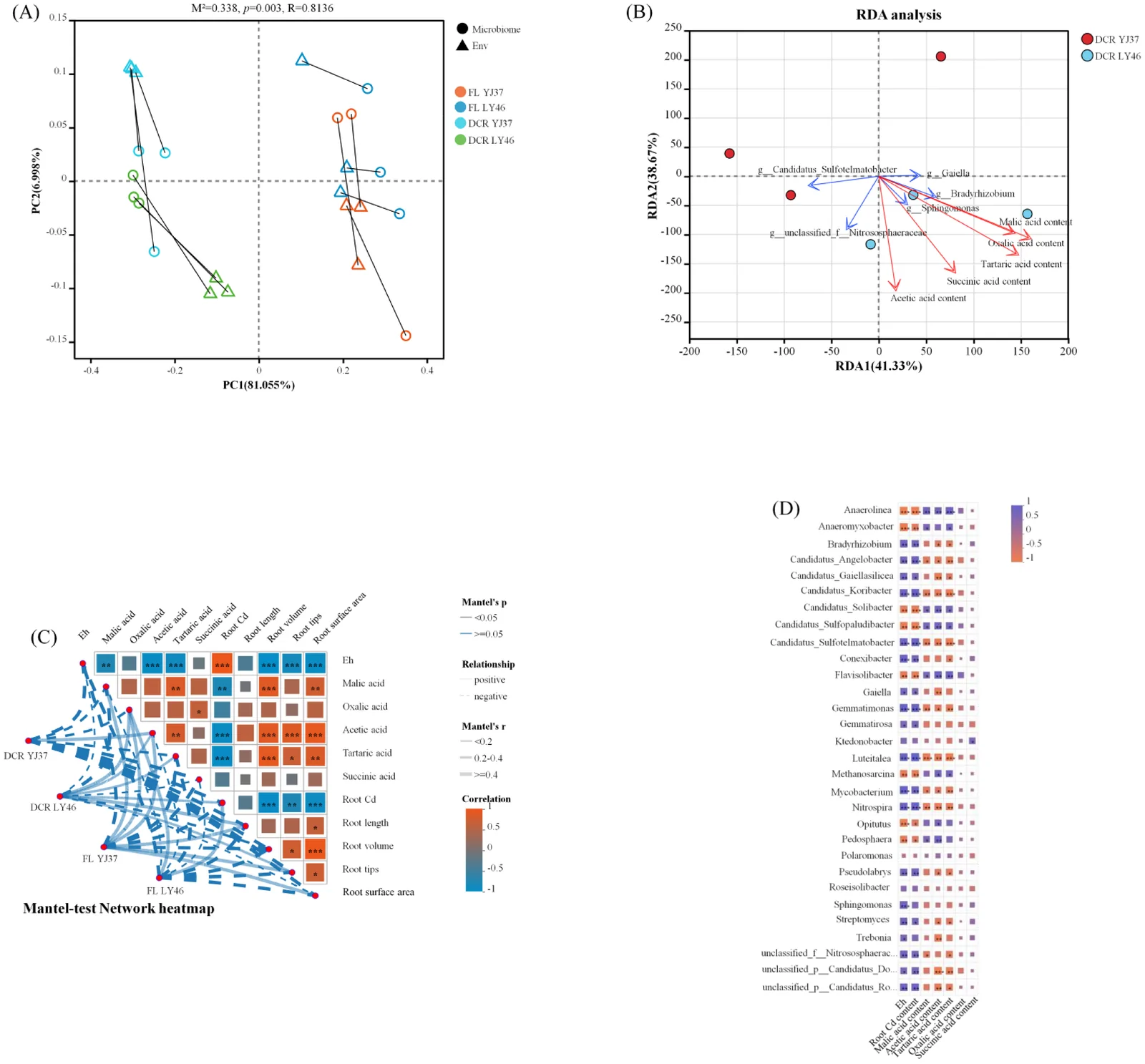
Relationship between environmental factors and microorganisms **(A)** Procrustes analysis of the correlation between organic acids and rhizosphere bacteria **(B)** Correlation between rhizosphere microorganisms and low-molecular-weight organic acids under dryland rice cultivation conditions (at the genus level) **(C)** Mantel test of rhizosphere environmental factors and root physiological traits under different treatments **(D)** Cluster correlation heat map of bacterial community at the genus level with root Eh and low-molecular-weight organic acids. Bacteria are significantly correlated with low-molecular-weight organic acids: *p < 0.05, **p < 0.01.

Under different water conditions, the rhizosphere environmental factors of rice exhibited significant correlations with factors such as root low-molecular-weight organic acids (**Figure 7C**). Soil redox potential (Eh) is the core driving force for changes in rhizosphere environmental factors, showing a highly significant negative correlation with low-molecular-weight organic acids such as acetic acid and tartaric acid (*P* < 0.001), and also showing a highly significant positive correlation with cadmium accumulation in the roots. This confirms that water regulation promotes cadmium release and transport to rice root systems by changing the soil redox environment. At the same time, acetic acid and tartaric acid show a highly significant negative correlation with cadmium content in the roots (*P* < 0.001), and a positive correlation with morphological traits such as root length and root surface area, revealing a strong physiological synergistic effect between root development and secretion release. Mantel test results indicate that there are differentiated driving effects of different water and variety treatments on rhizosphere microenvironmental factors, with some environmental factors reaching a significant correlation level between treatments (Mantel’s *P* < 0.05, r ≥ 0.4). Among them, ‘LY46’ has a strong specific association with certain organic acid (such as succinic acid, oxalic acid) network branches, indicating that this variety plays a key role in actively reshaping the rhizosphere microenvironment, preventing cadmium from being transported to the aboveground parts by secreting organic acids and regulating root growth.

Eh has a significant positive correlation with cadmium content in rice roots, indicating that Eh is a key environmental factor affecting cadmium uptake in roots (**Figure 7D**). Malic acid, acetic acid, tartaric acid, and oxalic acid have a significant negative correlation with Eh and cadmium content in roots, and show a significant positive correlation with obligate or facultative anaerobic bacteria such as *Anaerolinea*, *Anaeromyxobacter*, *Methanosarcina*, etc., and have a significant negative correlation with genera such as *Gemmatimonas*, *Nitrospira*, Candidatus_*Koribacter*, etc. This indicates that low redox potential drives the enrichment of specific microorganisms (such as anaerobic bacteria like *Anaerolinea*) and is accompanied by a significant increase in the content of organic acids such as malic acid, acetic acid, tartaric acid, and oxalic acid; the accumulation of these organic acids may play a role in fixing cadmium or inhibiting cadmium uptake, leading to a significant decrease in cadmium content in plant roots. Conversely, under high Eh environments, organic acids decrease, microbial structure changes, and cadmium uptake in roots intensifies.

## 4 Discussion

### 4.1 The effect of water regulation on soil Eh, root low-molecular-weight organic acids, and cadmium absorption in rice

Water resource scarcity has become the main limiting factor for rice production, and rice dryland technology is of great significance for water conservation and grain production. Compared with flooding conditions, the Cd content in the two rice root systems increases under dryland conditions (**Figure 2B**). The study by Yang et al. (2021) shows that continuous flooding can reduce Cd accumulation in rice plants. Lower redox potential is conducive to the fixation of Cd in paddy soil. During the flooding process, iron reduction promotes the formation of amorphous iron oxides, providing adsorption sites for Cd; with the enhancement of reduction, sulfate reduction promotes the precipitation of Cd sulfides (Qin et al., 2022). Continuous flooding also reduces the absorption and accumulation of Cd by rice by lowering the soil available Cd content (Hu et al., 2013).

Water conditions can affect soil Eh, and soil Eh is an important indicator of soil aeration, with soil aeration being positively correlated with Eh. In this study, under dryland conditions, the maximum Eh was 229.67 mV, and the soil was in an oxidized state. Under flooding conditions, the minimum Eh was -137.33 mV (**Figure 2A**), and the soil was in a reduced state. After flooding, the soil reductivity increased, and the longer the flooding duration, the lower the Eh, and the water-soluble Cd in the rhizosphere soil solution also rapidly decreased. Eh is significantly correlated with cadmium content in rice roots, indicating that Eh is the most critical factor affecting Cd levels in rice plants (Liu et al., 2022). However, Xu et al. (2021) found that water management increased the Eh in the rhizosphere of low-Cd rice varieties and reduced their absorption of Cd. Soil Eh is an important factor affecting the availability and chemical form of soil Cd.

Root exudate composition is complex, including inorganic ions, protons, and various organic compounds, which are secreted and released by different segments of the root during plant growth and are inherent physiological characteristics of the root (Guo et al., 2020). The organic acids secreted by plant roots change under Cd stress, and the concentration of organic acids after Cd stress also changes for different rice varieties, reflecting the plant’s response to Cd. Low-molecular-weight organic acids secreted by plant roots can form soluble complexes with metal ions, thereby affecting the mobility, solubility, and bioavailability of metals in the soil (Bali et al., 2020). In the present study, compared with flooded conditions, dryland conditions significantly reduced the contents of tartaric acid, acetic acid, and malic acid in dryland rice (**Figure 3**). This aligns with the findings of Liang et al. (2020), who reported that shifting from continuous flooding to dryland conditions (-30 kPa) decreased the concentrations of citric acid, malic acid, oxalic acid, tartaric acid, and total organic acids in rice roots. Under dryland conditions, the Cd content in rice roots was significantly higher than that under flooded conditions. Furthermore, the root exudation of organic acids in ‘LY46’ was higher than in ‘YJ37’, while the root Cd content in ‘LY46’ was lower than in ‘YJ37’. This increase in organic acid levels, accompanied by a decrease in Cd accumulation, may represent an avoidance mechanism employed by plants to maintain Cd concentrations below toxic thresholds (Song et al., 2025). Malic acid exuded by ‘LY46’ roots can form chelates with Cd^2+^, regulating intracellular Cd speciation and vacuolar compartmentalization, thereby mitigating the direct toxicity of free Cd^2+^ to cellular metabolism (Guo et al., 2017). Oxalic, succinic, and malic acids significantly reduce the concentration of available Cd in both the environment and plant tissues by promoting Cd adsorption and immobilization on rhizosphere particles (Yu et al., 2022). The decrease in acetic acid content under dryland conditions compared with flooded conditions is also consistent with the study by Lu et al. (2021), where Cd accumulation in sunflower roots increased with higher concentrations of applied acetic acid. These results indicate that low-molecular-weight organic acids (LMWOAs) exuded by roots act as a key factor in altering Cd speciation, and the reduced LMWOA levels in the roots of both rice cultivars under dryland conditions decrease soil Cd activation. Plants exude LMWOAs to chelate metal ions; these LMWOAs reflect the active defense capability of rice, which enhances the defense capacity of dryland rice by recruiting beneficial microorganisms. Root exudates play a vital role in plant-rhizosphere microbiome interactions, assisting plants in recruiting rhizosphere microbes.

### 4.2 Effects of water regulation under cadmium stress on the root microbial community structure of rice

Soil moisture conditions and low-molecular-weight organic acids can jointly drive the succession of the rhizosphere microbial community structure by directly altering the root microenvironment (Zhalnina et al., 2018). On one hand, soil moisture directly regulates the growth and metabolic processes of microorganisms; on the other hand, moisture affects the availability and mobility of dissolved organic matter, thereby influencing the respiratory substrates and energy supply available to microorganisms (Francioli et al., 2021). In this study, the moisture conditions had a highly significant effect on the content of low-molecular-weight organic acids. Under flooding conditions, the contents of tartaric acid, acetic acid, malic acid, and oxalic acid were significantly higher than under dryland cultivation. At the same time, the soil redox potential was significantly elevated under dryland conditions, and the differences in organic acid secretion patterns, together with the differentiated rhizosphere chemical environment, shaped the differences.

Different water conditions further shaped the structural differences of the rhizosphere microbial community by altering the soil redox state (**Figure 4**). Under dryland conditions, the reduction in microbial community diversity suggests that the toxicity screening effect of heavy metal Cd eliminated sensitive microorganisms, resulting in a sharp decline in rhizosphere community diversity; however, it stimulated the specific enrichment of tolerant strains such as *Pseudomonas* and *Bacillus*. Rhizosphere microbes play a crucial role in enhancing or controlling the uptake of heavy metals by crops and in enhancing environmental adaptability. The rhizosphere bacterial community of crops with low cadmium accumulation has higher diversity and richness, which helps alleviate heavy metal stress and maintain crop health. The roots of low cadmium-accumulating plants usually accumulate beneficial bacterial communities that support plant growth and development. Many bacterial species, such as Pseudomonas, exhibit high cadmium ion fixation ability and strong cadmium tolerance (Ali et al., 2022). *Acidobacteriota* are beneficial microorganisms that promote plant growth and enhance their defense capabilities when plants are subjected to abiotic stress (Kielak et al., 2016). Under cadmium stress, shifts in the rice microbial community across different water regimes foster plant growth, enhance tolerance, and diminish cadmium accumulation. Under flooding conditions, *Pseudomonadota*, *Bacteroidota*, and *Bacillota* Firmicutes are the dominant phyla in the study, which have the effect of promoting rice growth and reducing cadmium content in rice roots. The anaerobic Myxococcus genus, enriched in rice roots under flooding conditions, reduces the bioavailability of cadmium by activating phosphorus, thereby reducing cadmium accumulation (Zhang et al., 2025). Under dryland conditions, the *Nitrospirota* enriched in rice roots participates in regulating the carbon-nitrogen cycle (e.g., CO_2_ fixation and nitrite oxidation), which may change the cadmium speciation (e.g., promoting the solubility of Cd^2+^), thereby affecting plant uptake (Zhang et al., 2023). Studies have shown that *Sphingomonas* sp. can reduce the Cd exchangeable content in the rhizosphere of rice at maturity by 27%, significantly reducing plant Cd accumulation (Cheng et al., 2021); *Bradyrhizobium* sp. *NLH25* can adsorb and intracellularly accumulate a large amount of Cd (Bianucci et al., 2011); the *Gaiella* genus has potential effects in the prokaryotic carbon fixation pathway (Wei et al., 2024). Under cadmium stress, changes in the microbial community can protect plant growth, enhance tolerance, and reduce cadmium accumulation in plants.

### 4.3 Rice modulates low-molecular-weight organic acids in roots to recruit beneficial microorganisms

Plant water status is an important factor influencing root exudation (Vives-Peris et al., 2020). Compared with flooded conditions, plants under aerobic cultivation are more susceptible to severe cadmium (Cd) stress; root damage weakens organic acid uptake, leading to increased root organic acid contents. The anions dissociated from these organic acids can not only balance the increased intracellular metal ion concentrations but also regulate cellular pH and osmotic pressure, thereby helping plants cope with the combined stresses of drought and Cd (Magdziak et al., 2025). Under flooded conditions, the soil redox potential (Eh) is markedly reduced, converting free Cd²^+^ into highly insoluble CdS precipitates. Meanwhile, flooding induces a significant increase in organic acid secretion from rice roots and controls microbial competition by regulating carbon (C) supply or releasing inhibitory compounds (Maurer et al., 2021), thereby significantly reducing root Cd content. Under aerobic conditions, however, enhanced soil aeration markedly increases Eh, resulting in a high-Eh oxidative state, which causes oxidative decomposition of cadmium sulfide and promotes Cd desorption from iron oxides; consequently, Cd²^+^ activity increases sharply, and Cd content in rice roots increases.

Under aerobic conditions, the organic acid content of ‘LY46’ was significantly higher than that of ‘YJ37’. Low-molecular-weight organic acids such as oxalic acid, succinic acid, and acetic acid form chelates with Cd²^+^, directly preventing root uptake (Huang et al., 2023). At the same time, as specific signaling molecules and nutrients, these organic acids induced chemotactic responses (Meng et al., 2022), precisely driving and enriching beneficial microbial groups such as *Sphingomonas*, *Bradyrhizobium*, and unclassified_f_*Nitrososphaeraceae* (*P* < 0.05). Under Cd stress, the directionally recruited *Pseudomonas* utilized organic acids as carbon sources for extensive colonization and secreted large amounts of exopolysaccharides (EPS) to form biofilms, thereby physically adsorbing and biologically precipitating residual Cd²^+^ in the rhizosphere and preventing its entry into roots (Zhao et al., 2025), thereby reducing root cadmium content. Microorganisms such as *Bradyrhizobium* and *Sphingomonas* enhanced rice’s physiological tolerance to Cd and drought through biosorption, intracellular accumulation, and improved rhizosphere microenvironment. In addition, the *Chloroflexi* and *Actinobacteriota* were enriched under aerobic cultivation, owing to their relatively thick cell walls, protected cellular integrity, and reduced water loss (Xu et al., 2018a). In summary, this study elucidated the regulatory effects of different water management regimes on root organic acids and rhizosphere microorganisms of aerobic rice under Cd stress, revealed the rhizosphere ecological adaptation mechanisms, and provided new strategies and methods for agroecological regulation and soil health management, as well as a theoretical basis for further research on rice root environment and microbial interaction mechanisms.

## 5 Conclusion

Under dryland cultivation, rice root surface area, root volume, and root tip number were all significantly suppressed, soil redox potential (Eh) was significantly elevated, and root Cd accumulation in rice was significantly increased compared with the flooding treatment. Compared with continuous flooding, dryland treatment overall decreased the contents of tartaric acid, acetic acid, and malic acid in rice roots, and reduced the diversity and richness of the rhizosphere soil microbial community.

Under flooded conditions, anaerobic genera (such as *Anaeromyxobacter* and *Pedosphaera*) predominated; whereas under dryland conditions, Cd-tolerant aerobic/facultative anaerobic genera (such as *Sphingomonas*, *Bradyrhizobium*, and *Gaiella*) were significantly enriched. Under dryland stress, the exudation amounts of succinic acid, tartaric acid, malic acid, and oxalic acid of ‘LY46’ were significantly higher than those of ‘YJ37’. Organic acids can directly chelate free Cd^2+^ in the rhizosphere and simultaneously serve as signaling molecules to directionally recruit beneficial bacterial communities such as *Sphingomonas* and *Bradyrhizobium*, alleviating Cd toxicity through extracellular immobilization and bioaccumulation. In summary, water regulation jointly regulates Cd uptake and accumulation in dryland rice through a synergistic mechanism involving altering soil Eh, driving root low-molecular-weight organic acid exudation, and reshaping rhizosphere microbial communities, providing a theoretical basis for the safe production of dryland rice.

## Data Availability

The original contributions presented in the study are publicly available. These data can be found here: https://www.ncbi.nlm.nih.gov/bioproject/PRJNA1524109.
